# Serotonin 5-HT6 receptors affect cognition in a mouse model of Alzheimer’s disease by regulating cilia function

**DOI:** 10.1186/s13195-017-0304-4

**Published:** 2017-09-20

**Authors:** Lili Hu, Bingjie Wang, Yan Zhang

**Affiliations:** State Key Laboratory of Biomembrane and Membrane Biotechnology, College of Life Sciences, PKU-IDG/McGovern Institute for Brain Research, Peking University, Room 239, Beijing, 100871 China

**Keywords:** 5-HT6, Cilia, Axon, Axon initial segment, Alzheimer’s disease

## Abstract

**Background:**

Serotonin receptor 5-HT6 is involved in cognition and Alzheimer’s disease (AD) development. However, the mechanism of 5-HT6 in AD pathology is not clear.

**Methods:**

Since 5-HT6 is almost exclusively expressed in the primary cilia, using immunostaining we examined the number of cilia in the hippocampus of AD animal model APP/PS1 mice. By overexpressing and knocking down 5-HT6 in the primary cultured hippocampal neurons, we investigated the roles of 5-HT6 in alternating ciliary morphology. Furthermore, 5-HT6 antagonist was applied to confirm its roles in cognition using the Morris water maze test, Y maze, and fear conditioning.

**Results:**

In the present study, we found that the primary cilia were elongated in the hippocampus of APP/PS1 mice compared with WT mice. 5-HT6 regulated cilia length, influenced cilia and axon initial segment (AIS) morphology, and affected localization of ARL13B and AnkG. We also found that, by changing cilia morphology, the AIS was elongated, branched, and more proximal to the cell body in both WT and APP/PS1 mouse neurons. Alterations of cilia also decreased the axonal length in WT and APP/PS1 neurons. Furthermore, in the water maze test, Y maze, and fear conditioning test, 5-HT6 antagonist SB271046 recovered the cognitive impairment of APP/PS1 mice.

**Conclusion:**

We suggest that 5-HT6 plays a critical role in AD development through regulating the morphology and function of neuronal primary cilia, which is possibly related to the AIS and axon alterations in AD development.

**Electronic supplementary material:**

The online version of this article (doi:10.1186/s13195-017-0304-4) contains supplementary material, which is available to authorized users.

## Background

Alzheimer’s disease (AD) is the most common form of dementia in people aged 65 years and older. AD is associated with impairments in memory, cognition, language, behavior, and personality [1]. The pathology of AD in the brain includes extracellular senile plaques composed of amyloid beta (Aβ), intracellular tau aggregates known as neurofibrillary tangles (NFT), and neuronal and synaptic loss [1]. Many studies have shown that individuals with the 267C allele of 5-HT6 have increased risk of AD [2, 3]. Several studies have reported that 5-HT6 has great impact in cognition, especially in AD models [4]. Up to now, there is no effective treatment for AD. During the past 30 years, many studies have indicated that 5-HT6 may be a potential target for cognitive improvement in AD [5]. Some 5-HT6 antagonists are currently being tested in clinical trials [6]. However, although there is a strong link between 5-HT6 and AD development and treatment, the mechanism of how 5-HT6 is associated with AD is still not well understood. Besides, several 5-HT6 antagonist drugs, such as Pfizer's SAM-760 and SAM-531, Abbott's A-964324, Avineuro Pharmaceuticals' AVN-322, Suven's SUVN-502, Synosia Therapeutics' SYN-120, idalorpidine, and others, showed no significant positive effects for AD patients [7]. The clinical failures made the pathophysiology of 5-HT6 antagonist more uncertain and indicated the necessity to clarify the inner function of 5-HT6 in AD. In the present study, we aimed to determine the mechanism of 5-HT6 in AD pathology.

Previous studies have shown that 5-HT6 can influence dendritic growth and neuronal migration [8, 9], suggesting that 5-HT6 might influence neuritic and axonal development. Furthermore, it has been reported that AD is associated with axonal abnormalities, including axonal swelling and abnormal accumulation of axonal proteins, which are related to dystrophic neurites and amyloid plaque formation [10]. Abundant age-dependent axonal spheroids and myelin ovoids have been reported in the spinal cord of APP_Swe/London_/PS1_M146V_ mice, an established familial AD (FAD) transgenic mouse model [11]. However, the causative association of axonopathy to AD and the pathways involved in AD-related axonal abnormalities are not defined. The study of 5-HT6 promotes research on axonal abnormalities in AD. Furthermore, a previous study indicated that BBS8, a component of the primary cilia, influences axon targeting [12]. The hedgehog signaling pathway, which is active in primary cilia, regulates axonal length in hippocampal neurons [13]. Therefore, the study of 5-HT6 in APP/PS1 mice would contribute to exploring the relationship between primary cilia and axons.

5-HT6 is highly expressed in the central nervous system (CNS) [6, 14]. High levels of 5-HT6 expression have also been reported in the striatum, olfactory tubercle, nucleus accumbens, cortex, and hippocampus [14, 15]. 5-HT6 mRNA is exclusively expressed in neurons [6]. In neurons, 5-HT6 is especially localized at the primary cilia [16, 17]. Primary cilia are protrusions of the cell membrane that are approximately 1–5 μm in length and anchored to the ciliary basal body (a modified mother centriole) [18]. Cilia play critical roles in signaling in the neuronal system. For example, in the absence of the hedgehog signaling pathway ligand sonic hedgehog (shh), Patched receptors located at the cilia prevent Smoothened receptors from entering the cilia. In the presence of shh, Patched receptors move away from the cilia, allowing Smoothened receptors to accumulate in the cilia [19, 20]. Various human diseases are related to ciliary dysfunction, including cystic disease of the kidney, polydactyly, brain malformations, hydrocephalus, blindness, anosmia, obesity, and cognitive deficits [21]. However, the functions of primary cilia in neurons and neuronal diseases, such as neurodegenerative diseases including AD, are largely unknown. Since 5-HT6 is mainly localized in the cilia, we hypothesized that 5-HT6 plays important roles in AD by regulating ciliary function.

## Methods

### Plasmids, chemicals, and antibodies

The mouse EGFP-tagged 5-HT6 plasmid was provided by Kirk Mykytyn (Ohio State University, USA). We constructed 13 mutagenesis plasmids. In each clone, the codons for amino acids 69, 70, 72, 106, 230, 234, 262, 265, 284, and 350 were replaced by triplet nucleotides. Mouse GFP-tagged ARL13B was amplified from cDNA from the mouse brain. 5-HT6-mcherry plasmid was constructed. PEGFP-N3 and mcherry-N3 were purchased from Addgene.

SB271046 drug (Santa Cruz Biotechnology), 5-HT6R siRNA (QIAGEN), siRNAs of 5-HT1A, 5-HT1B, 5-HT1D, 5-HT1F, 5-HT2A, 5-HT2B, 5-HT2C, 5-HT3A, 5-HT4, 5-HT5A, 5-HT5B, and 5-HT7 (Santa Cruz Biotechnology), 5-HT6R antibody (Abcam), MAP2 antibody (Abcam), AC3 antibody (Santa Cruz Biotechnology), SSTR3 antibody (Santa Cruz Biotechnology), ARL13B antibody (NeuroMab), ankyrin G antibody (Invitrogen), neurofascin 186 antibody (Abcam), γ-tubulin antibody (Sigma), IFT88 antibody (Proteintech), calbindin antibody (Swant), CamkII antibody (Millipore), Gad antibody (Abcam), Nav1.1 antibody (NeuroMab), Nav1.6 antibody (Alomone), Nav1.2 antibody (NeuroMab), Kv1.1 antibody (Alomone), and AlexaFluor fluorescence antibodies (Invitrogen) were utilized.

### Cell culture and transfection

Primary culture neurons were cultured from the hippocampus of newborn C57 and APP/PS1 mice. The use of mice was in accordance with the regulations of the Peking University Animal Care and Use Committee (LSC-ZhangY-1). Fetal hippocampal samples were dissociated from the brain and digested by 0.25% trypsin (Invitrogen). Digestion was stopped by adding DMEM-F12 medium (Gibco) with 10% FBS (Gibco), after which the tissue was dispersed by a pipettor. After 2 minutes of precipitation, the supernatant was collected and centrifuged at 500 × *g* for 2 minutes. The cells were resuspended in DMEM-F12 medium with 10% FBS and plated on a coverslip coated with poly-_D_-lysine (Sigma) in 5% circulating CO_2_. Neurobasal medium (Gibco) containing Pen-Strep (Invitrogen), B27 (Gibco), and GlutaMAX (Thermo Fisher) was added to the medium after 4 hours. Half of the medium was replaced with fresh medium every 3 days.

Calcium phosphate transfection was used to transfer siRNA or a plasmid into hippocampal neurons. Neurons were transfected after 3–8 days. A mixed solution containing plasmids, CaCl_2_, H_2_O, and HBS (pH 7.1) was prepared and centrifuged. After adding the mixture to each well for 1–1.5 hours, HBS (pH 6.8) was added to each well to clear the precipitate. Finally, the original medium was added to every well and the cells were placed in a 37 °C incubator.

### Immunostaining

Cells were washed in PBS and fixed in 4% PFA (Sigma) at room temperature. Next, cells were permeabilized in 0.1% Triton at 4 °C, after which they were blocked in 5% donkey serum at room temperature and incubated with primary antibodies at 4 °C for 24 hours. Secondary antibodies were added to the cells for 1 hour in the dark. Nuclei were stained by DAPI (Sigma) for 15 minutes. Finally, coverslips with cells were put onto slides for imaging.

Each mouse was perfused with 0.9% saline and 4% PFA by myocardial perfusion. Brain slices were coated with OCT (Tissue-Tek) and sectioned by freezing microtomy into sections 35 μm thick. Next, the tissue samples were permeabilized in 0.3% Triton, blocked in 5% donkey serum, incubated in primary and secondary antibodies, and stained with DAPI. The brain slices were blocked in protection liquid.

### Coimmunoprecipitation

HEK293T cells were transfected using Lipofectamine 2000 (Invitrogen) for 48 hours. Cells were collected using RIPA lysis buffer (R&D) and proteins were obtained by centrifugation. Proteins were incubated with protein A + G agarose beads (Beyotime) and antibodies overnight. Immunoprecipitated proteins were collected after washing three times with PBS (Sigma). The bicinchoninic acid assay (Pierce) was used to measure protein concentration. Proteins were denatured at 100 °C for 5 minutes and separated in 10% SDS-PAGE at 80 mA for 2 hours. They were then transferred to PVDF membrane (Millipore) at 100 mA for 2 hours. The membrane was blocked in 5% BSA (Sigma) in TBS (Sigma) with 0.1% Tween 20 (TBST) at room temperature for 1 hour. The antibodies were diluted at suitable concentration and added into TBST with 5% BSA. Primary antibodies were incubated overnight at 4 °C. HRP-conjugated second antibodies were added after washing three times for 10 minutes by TBST. Enhanced chemiluminescence was used to detect optical density of HRP after washing three times for 10 minutes. BioRad ChemiDox (BioRad) was used to analyze optical density of HRP.

### Morris water maze test

Twelve-month-old male APP_swe_/PS1_ΔE9_ and C57 WT mice were subjected to the water maze test. Mice were kept at room temperature with food and water. Both APP/PS1 and WT mice were divided into two groups. Every group included 12 male APP/PS1 mice. The mice in the SB271046 group were given an intraperitoneal injection of SB271046 (10 mg/kg) 2 hours before water maze training and tested for 7 days. In the vehicle group, mice were injected with an equal amount of 0.9% NaCl for 7 continuous days.

The water maze test consisted of a circular pool (60 cm in diameter and 50 cm high). The pool was filled with 21–22 °C water at a depth of 30 cm. The hidden platform was 11 cm in diameter and placed 1.5 cm under the surface of the water. The pool was divided into four quadrants. The hidden platform was in one of the four quadrants. Mice were tested in the four quadrants with a fixed order. The starting location was random in the four quadrants. Mice spent four sessions of 90 s in the pool to adapt to the water environment 1 day before the experiment. The test began when a mouse was placed on the quadrants facing the wall and ended when the mouse got onto the platform. If a mouse got onto the platform within 90 s, it remained for 10 s on the platform. If the mouse failed to get onto the platform within 90 s, it was guided to the platform and stayed there for 10 s. The mice were trained and recorded in four quadrants per day for 6 continuous days to measure the swimming speed, time to the platform, and swimming distance with the hidden platform. On day 7, the hidden platform was removed. Then, we tested the time spent in the target quadrant without the platform and the crossing times at the original location of the platform on the seventh day. The position of the mouse was traced by the automatic tracking system (CSI) in real time.

### Y maze

Twelve-month-old male APP_swe_/PS1_ΔE9_ and C57 WT mice were used to test in the Y maze. APP/PS1 mice were divided into two groups (*n* = 12). One group was injected with SB271046 (10 mg/kg) and the other was injected with equal vehicle (0.9% NaCl). C57 WT mice were also divided into SB271046 and vehicle groups (*n* = 12). Drug or vehicle was injected 2 hours before the Y maze. The apparatus consisted of three arms which were 34 cm long, 8 cm wide, and 14.5 cm high. There are different images pasted on the end of three inner arms, containing a red circle, a black square, and a white triangle. The test began when a mouse was placed on the apparatus facing the wall. After 10 minutes, the experiment was ended. The automatic tracking system (CSI) traced the position of the mouse in real time and calculated the times when mice chose the correct arm. The correct arm was the one which was different form the last and next arms which mice chose.

### Fear conditioning

Twelve-month-old male APP_swe_/PS1_ΔE9_ and C57 WT mice were used. Both APP/PS1 and C57 WT mice were divided into two groups (*n* = 12). One group was injected with SB271046 (10 mg/kg) and the other was injected with equal vehicle (0.9% NaCl). Drug or vehicle was injected 2 hours before training or testing for 2 continuous days. Mice were adapted on an operant box for 6 minutes on the day before the experiment. Training started for a period of 6 minutes on day 1. Mice received a 60-dB noise for a period of 30 s, followed by a 0.8-mA foot shock for a period of 5 s. This stimulus was repeated three times with an interval of 150 or 90 s. The mice were then placed in the chamber for 5 minutes on day 2. The device then recorded the duration of the freezing time of the mice.

### Statistical evaluation

For univariate data analysis, data were detected whether they were normally distributed or not. If data were equally distributed, a *t* test was used in two groups and one-way ANOVA followed by Dunnett’s test were used in more than two groups. If data were nonnormally distributed, a Mann–Whitney *U* test was used in two groups and the Kruskal–Wallis test followed by Dunnett’s test were used in more than two groups. For multivariable data analysis, two-way ANOVA followed by Bonferroni test or three-way ANOVA followed by Holm–Sidak test were used.

## Results

### 5-HT6 regulated primary cilia length

**Fig. 1:**
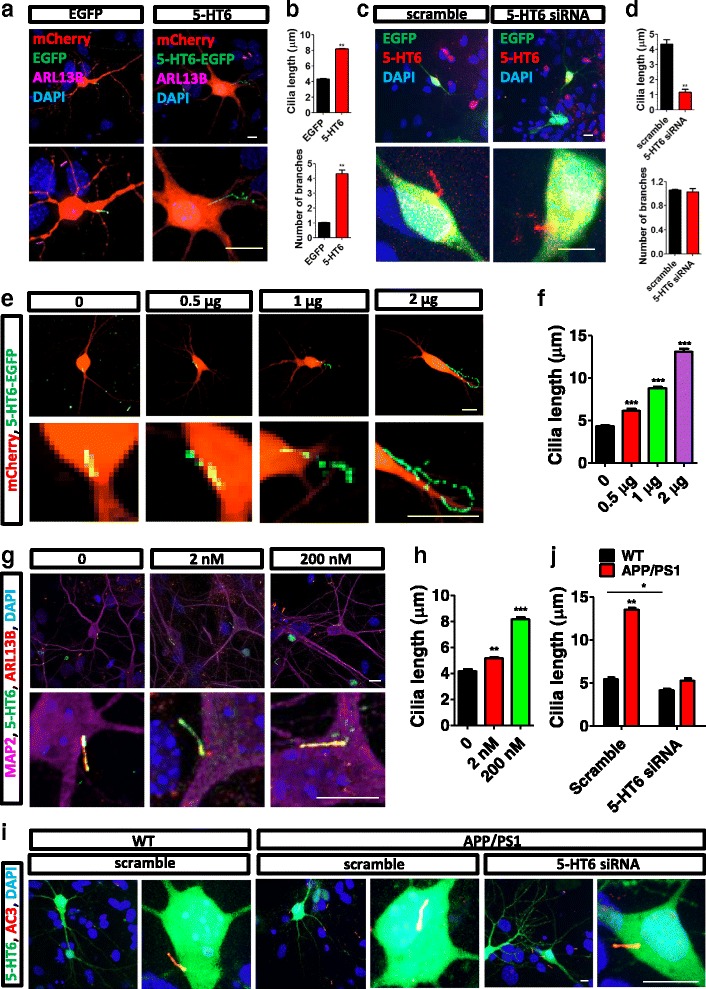
5-HT6 regulated the length of primary cilia in APP/PS1 mice. (**a**) Overexpression of 5-HT6 induced elongation and branched primary cilia in hippocampal neurons. 5-HT6 (green) and ARL13B (purple) indicated primary cilia. DAPI indicated nuclei. (**b**) Overexpression of 5-HT6 increased the length and branching numbers of primary cilia. Cilia length, **p < 0.01 (*t* test); number of branches, **p < 0.01 (Mann–Whitney *U* test). (**c**) Knocking down 5-HT6 decreased the length of primary cilia in hippocampal neurons. (**d**) Length of primary cilia was reduced by siRNA against 5-HT6. Cilia length, **p < 0.01 (Mann–Whitney *U* test). The branching number of primary cilia was unchanged (remaining at 1) after exposure to siRNA against 5-HT6. Number of branches, *p* = 0.62 (*t* test). (**e**) Primary cilia elongated gradually with increasing 5-HT6 transfection. 5-HT6 (green) indicated primary cilia. (**f**) Cilia length increased with upregulated 5-HT6. All groups were compared with the control group, which was transfected with no vectors. 0.5 μg, ***p < 0.001; 1 μg, ***p < 0.001; 2 μg, ***p < 0.001 (Kruskal–Wallis test, *p* < 0.001). (**g**) Primary cilia length increased as the 5-HT concentration increased. 5-HT6 (green) and ARL13B (red) were markers of primary cilia. MAP2 (purple) served as a neuronal marker. (**h**) Ciliary length was elongated by 5-HT in a gradual and concentration-dependent manner. All groups were compared with the control group to which was applied 0 nM 5-HT. 2 nM, **p < 0.01; 200 nM, ***p < 0.001 (one-way ANOVA, *F*_2,121_ = 220.6, *p* < 0.001). (**i**) siRNA against 5-HT6 decreased the primary ciliary length of hippocampal neurons in APP/PS1 mice. AC3 (red) indicated primary cilia. (**j**) Knocking down 5-HT6 reduced the ciliary length of hippocampal neuron in APP/PS1 and WT mice. All groups were compared with the group exposed to scrambled siRNA. siRNA versus Scramble in WT, *p < 0.05; siRNA versus Scramble in APP/PS1, **p < 0.01 (two-way ANOVA: interaction, *F*_1,195_ = 224.8, *p* < 0.001; treatment, *F*_1,195_ = 417.6, *p* < 0.001; gene, *F*_1,195_ = 389.6, *p* < 0.001). Scale bars, 10 μm. All data presented as mean ± SEM (≥3 independent experiments). 5-HT6, serotonin 6 receptor, DAPI 4',6-diamidino-2-phenylindole, EGFP enhanced green fluorescent protein

5-HT6 is mainly located at the primary cilia, where it plays an important role [22]. Our results confirmed that 5-HT6 was localized to the primary cilia of hippocampal neurons, colocalized with two other cilia-specific proteins ARL13B and SSTR3 (Additional file 1: Figure S1A). Exogenous 5-HT6-enhanced green fluorescent protein (EGFP) was also located at the cilia, which were marked by ARL13B, γ-tubulin, and intraflagellar transport protein 88 (IFT88) (Additional file 1: Figure S1B). Therefore, we speculated that 5-HT6 may be involved in AD pathogenesis via regulating cilia function. Overexpression of 5-HT6 increased the ciliary length of WT C57 mouse hippocampal neurons (Fig. 1a, b). In addition, overexpression of 5-HT6 increased the number of cilia branches (Fig. 1a, b). The cilia became shorter after 5-HT6 was knocked down by its siRNA (Fig. 1c, d). Also, we tested the effect of other siRNA of 5-HT receptors on cilia length, including 5-HT1A, 5-HT1B, 5-HT1D, 5-HT1F, 5-HT2A, 5-HT2B, 5-HT2C, 5-HT3A, 5-HT4, 5-HT5A, 5-HT5B, 5-HT6, and 5-HT7. Both siRNAs of 5-HT6 and 5-HT3A decreased cilia length (Additional file 2: Figure S2A, B). 5-HT6 regulated cilia length in excitatory and inhibitory neurons. Overexpression of 5-HT6 increased cilia length, but siRNA of 5-HT6 decreased cilia length in excitatory neurons (Additional file 3: Figure S3A, B). Similar results were found in inhibitory neurons (Additional file 3: Figure S3C, D). Furthermore, ciliary length was measured as 5-HT6 expression was increased gradually in hippocampal neurons, confirming that enhanced 5-HT6 expression was associated with cilia elongation (Fig. 1e, f). Moreover, ciliary length increased as the concentration of 5-HT, a typical agonist of 5-HT6, was increased (Fig. 1g, h).

### The primary cilia were elongated in the hippocampus of APP/PS1 mice

**Fig. 2:**
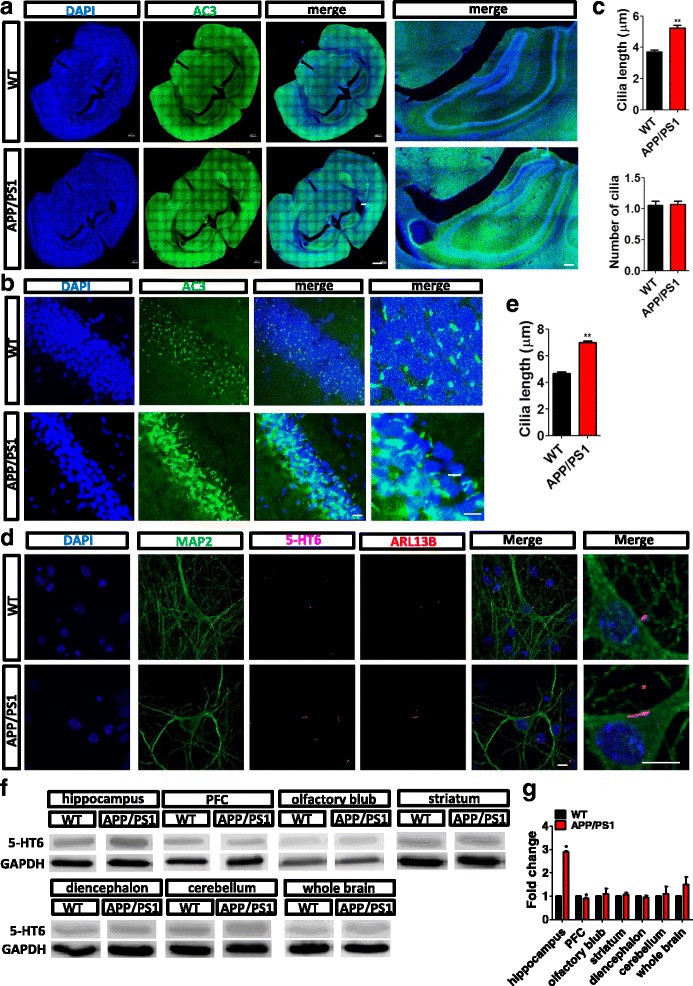
Elongated primary cilia and upregulated 5-HT6 in the hippocampus of APP/PS1 mice. (**a**) Primary cilia marker AC3 (green) indicated the primary cilia in brain sections from APP/PS1 and WT C57 mice by immunofluorescence staining in vivo. DAPI (blue) was used to indicate cell nuclei. Scale bars, 500 μm. (**b**) Primary cilia were elongated in the hippocampus of APP/PS1 mice. Scale bars, 10 μm. (**c**) Primary cilia of the hippocampus were obviously elongated in the hippocampus of APP/PS1 mice. Cilia length, **p < 0.01 (*t* test). The number of primary cilia (normalized to the number of cell nuclei) was similar in the hippocampus of APP/PS1 and WT mice. Number of cilia, *p* = 0.76 (Mann–Whitney *U* test). (**d**) Primary cultures of hippocampal neurons showed that the length of primary cilia was increased by immunostaining in vitro. ARL13B (red) and 5-HT6 (purple) indicated primary cilia. MAP2 (green) was used as a neuronal marker. DAPI (blue) indicated cell nuclei. (**e**) Primary cilia were elongated in primary cultures of hippocampal neurons from APP/PS1 and WT mice. Cilia length, **p < 0.01 (*t* test). (**f**) Protein levels of 5-HT6 and GAPDH in the hippocampus, PFC, olfactory blub, striatum, diencephalon, cerebellum, and whole brain of APP/PS1 and WT mice. GAPDH was used as an internal control. (**g**) In APP/PS1 mice, the protein level of 5-HT6 (normalized to GAPDH) was significantly increased in the hippocampus. Hippocampus, *p < 0.05; PFC, *p < 0.05; olfactory blub, *p* = 0.62; striatum, *p* = 0.22; diencephalon, *p* = 0.57; cerebellum, *p* = 0.71; whole brain, *p* = 0.13 (Mann–Whitney *U* test). All data presented as mean ± SEM (≥3 independent experiments). 5-HT6, serotonin 6 receptor, DAPI 4',6-diamidino-2-phenylindole

We first measured the length of cilia in cultured APP/PS1 and WT mouse neurons. APP/PS1 neurons had increased ciliary length. siRNA of 5-HT6 decreased cilia length in hippocampal neurons of APP/PS1 and WT mice (Fig. 1i, j). To measure the length of the primary cilia in vivo, brain slices of APP/PS1 and WT mice (6 months old) were prepared using cryosectioning and immunostaining. Adenylate cyclase 3 (AC3) and 4′,6-diamidino-2-phenylindole (DAPI) were used to label the primary cilia and indicate the nuclei, respectively. The primary cilia were elongated in the hippocampus of APP/PS1 mice (Fig. 2a–c). The number of cells with cilia, which was normalized by the number of nuclei, was similar in the APP/PS1 and WT mice (Fig. 2c). To verify elongation of the cilia of APP/PS1 mice in vitro, hippocampal neurons of newborn mice were cultured and stained for cilia markers. ADP-ribosylation factor-like protein 13B (ARL13B) was used to indicate the primary cilia [23]. The data confirmed that the length of the primary cilia of hippocampal neurons from APP/PS1 mice increased significantly in comparison with that of WT mice (Fig. 2d, e). Thus, both in-vivo and in-vitro experiments showed that the cilia of the hippocampal neurons of APP/PS1 mice were elongated.

5-HT6 expression levels were detected by western blot analysis in different brain sections from 6-month-old APP/PS1 or WT mice (Fig. 2f). In the olfactory blub, striatum, diencephalon, and cerebellum of APP/PS1 mice, the expression level of 5-HT6 protein was not significantly different from that of WT mice. However, expression levels of 5-HT6 in APP/PS1 mice was remarkably upregulated in the hippocampus and in comparison with WT mice (Fig. 2g). Therefore, we suggested that cilia elongation in APP/PS1 mice might be induced by upregulation of 5-HT6 levels.

### 5-HT6 regulated cilia morphology and protein localization

**Fig. 3:**
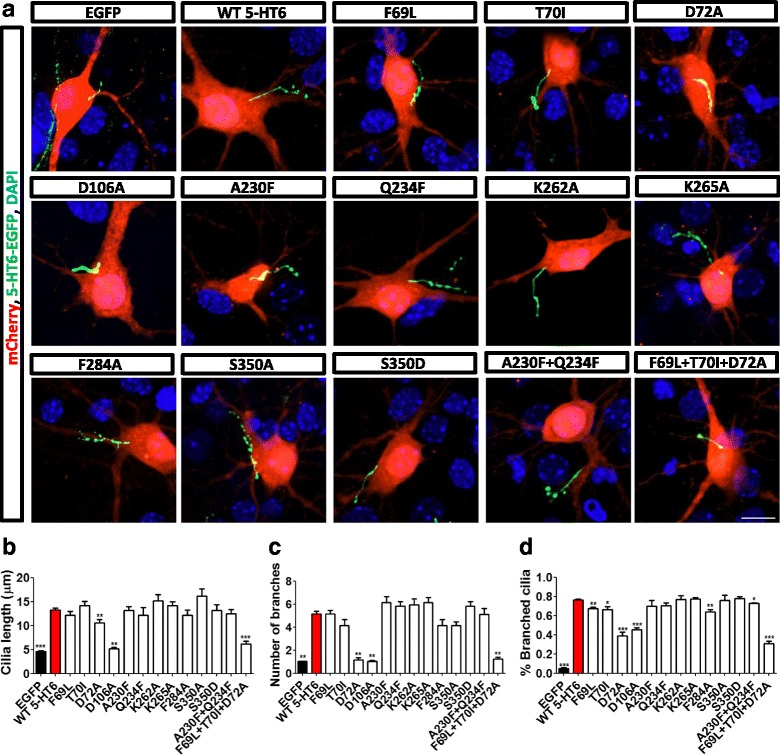
5-HT6 mutations regulated ciliary length, cilia branching number, and percentage of neurons with branched cilia. (**a**) D72A, D106A, and F69L + T70I + D72A mutations influenced ciliary length. These mutations of 5-HT6 restored primary ciliary length increased by overexpression of 5-HT6. 5-HT6 (green) indicated primary cilia. (**b**) D72A, D106A, and F69L + T70I + D72A mutations restored normal cilia length. EGFP, ***p < 0.001; D72A, **p < 0.01; D106A, **p < 0.01; F69L + T70I + D72A, ***p < 0.001 (Kruskal–Wallis test, *p* < 0.001). (**c**) D72A, D106A, and F69L + T70I + D72A mutations influenced the number of ciliary branches. These mutations of 5-HT6 significantly reduced the number of ciliary branches. EGFP, **p < 0.01; D72A, **p < 0.01; D106A, **p < 0.01; F69L + T70I + D72A, **p < 0.01 (Kruskal–Wallis test, p < 0.001). (**d**) F69L, T70I, D72A, D106A, F284A, A230F + Q234F, and F69L + T70I + D72A mutations decreased the percentage of branched cilia. EGFP, ***p < 0.001; F69L, **p < 0.01; T70I, *p < 0.05; D72A, ***p < 0.001; D106A, ***p < 0.001; F284A, **p < 0.01; A230F + Q234F, *p < 0.05; F69L + T70I + D72A, ***p < 0.001 (Kruskal–Wallis test, *p* < 0.001). All groups were compared with the 5-HT6 group, which was transfected with 5-HT6-GFP. Scale bars, 10 μm. All data presented as mean ± SEM (≥3 independent experiments). 5-HT6, serotonin 6 receptor, DAPI 4',6-diamidino-2-phenylindole, EGFP enhanced green fluorescent protein

To identify the key sites of 5-HT6 involved in its regulation of ciliary length, 13 reported mutations of 5-HT6 (F69L, T70I, D72A, D106A, A230F, Q234F, K262A, K265A, F284A, S350A, S350D, A230F + Q234F, and F69L + T70I + D72A) were examined. The related sequence information is presented in Additional file 4: Figure S4B. Mutations D72A, D106A, and F69L + T70I + D72A restored the ciliary length and the number of primary cilia branches when overexpressed in WT neurons (Fig. 3a–c and Additional file 4: Figure S4A). Mutations F69L, T70I, D72A, D106A, F284A, A230F + Q234F, and F69L + T70I + D72A restored the percentage of neurons with branched cilia (Fig. 3d). These results indicated that sites D72 and D106 and the combination of F69, T70, and D72 were primarily responsible for ciliary regulation by 5-HT6.

**Fig. 4:**
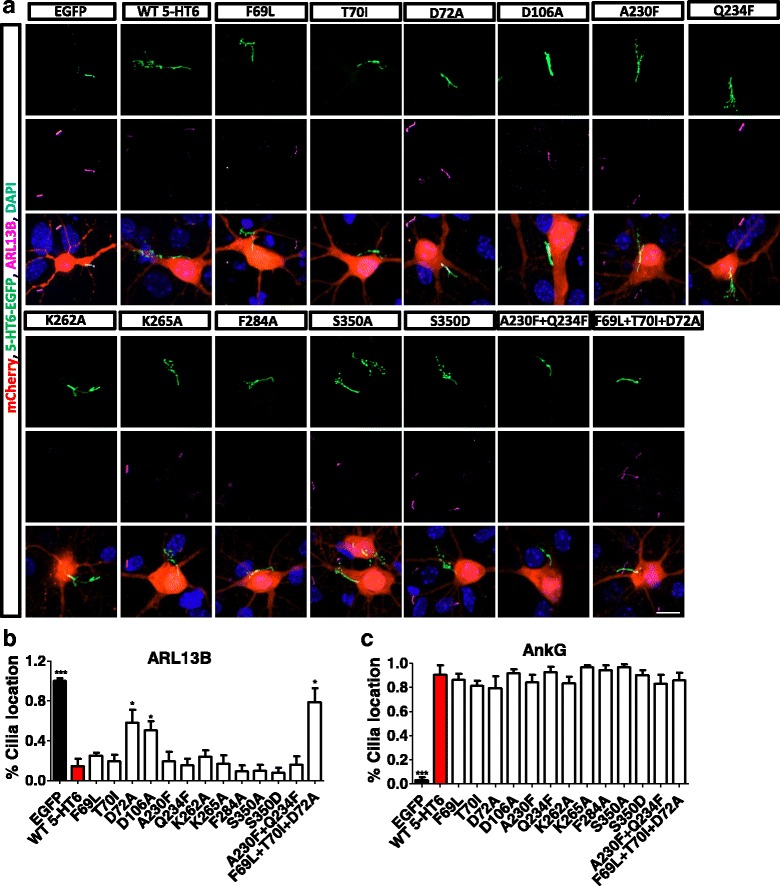
5-HT6 mutations regulated the locations of ARL13B and AnkG proteins. (**a**) D72A, D106A, and F69L + T70I + D72A mutations influenced the ciliary location of ARL13B. Overexpression of 5-HT6 delocalized ARL13B in primary cilia. D72A, D106A, and F69L + T70I + D72A mutations of 5-HT6 led to relocation of ARL13B (red) into the cilia. 5-HT6 (green) indicated primary cilia of hippocampal neurons. (**b**) D72A, D106A, and F69L + T70I + D72A mutations restored the ciliary location of ARL13B. Overexpression of 5-HT6 led to delocalization of ARL13B in primary cilia. EGFP, ***p < 0.001; D72A, *p < 0.05; D106A, *p < 0.05; F69L + T70I + D72A, *p < 0.05 (Kruskal–Wallis test, *p* < 0.001). (**c**) AnkG was originally located at the AIS, but it was located at the primary cilia following overexpression of 5-HT6. 5-HT6 mutations did not restore the location of AnkG. EGFP, ***p < 0.001 (Kruskal–Wallis test, *p* < 0.001). All groups were compared with the 5-HT6 group, which was transfected with 5-HT6-GFP. Scale bars, 10 μm. All data presented as mean ± SEM (≥3 independent experiments). 5-HT6, serotonin 6 receptor, DAPI 4',6-diamidino-2-phenylindole, EGFP enhanced green fluorescent protein

**Fig. 5:**
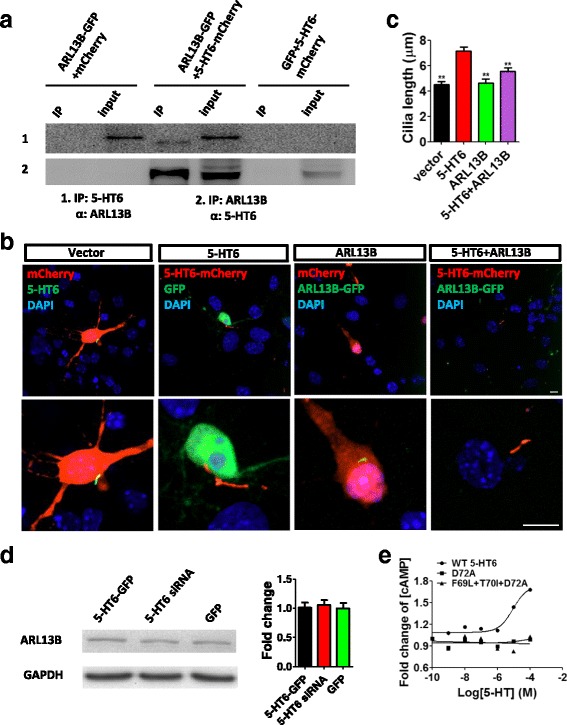
5-HT6 regulated primary ciliary length through ARL13B. (**a**) CO-IP showed that 5-HT6 could bind with ARL13B in Hek293T cells. Bidirectional results showed that 5-HT6 and ARL13B interacted. (**b**) ARL13B restored the length of primary cilia. Cotransfection of ARL13B and 5-HT6 reduced cilia length, which was increased by overexpression of 5-HT6. (**c**) Ciliary length was shortened by expression of ARL13B. All groups were compared with the 5-HT6 group. Vector, **p < 0.01; ARL13B, **p < 0.01; 5-HT6 + ARL13B, **p < 0.01 (Kruskal–Wallis test, *p* < 0.001). (**d**) 5-HT6 had no effect on the total protein level of ARL13B in HEK293T cells. Kruskal–Wallis test, *p* = 0.85. GAPDH was the internal control. (**e**) D72A and F69L + T70I + D72A mutations of 5-HT6 influenced the quantity of cAMP (two-way ANOVA: concentration, *F*_6,12_ = 5.45, *p* < 0.05; treatment, *F*_2,12_ = 9.31, *p* < 0.01). Scale bars, 10 μm. All data presented as mean ± SEM (≥3 independent experiments). 5-HT6, serotonin 6 receptor, DAPI 4',6-diamidino-2-phenylindole, GFP green fluorescent protein

In addition, we also found that 5-HT6 mutations altered cilia protein localization. ARL13B, normally located at the primary cilia, disappeared from the primary cilia with 5-HT6 overexpression (Fig. 4a). Mutations D72A, D106A, and F69L + T70I + D72A restored the ciliary location of ARL13B (Fig. 4b). 5-HT6 affected ARL13B localization at the primary cilia. Therefore, we speculated that ARL13B was involved in 5-HT6 regulation of ciliary length. CO-IP experiments indicated that ARL13B and 5-HT6 interacted in HEK293T cells (Fig. 5a). Anti-ARL13B antibodies were able to pull down 5-HT6 protein, while anti-5-HT6 antibodies were able to pull down ARL13B protein. An ARL13B-GFP vector was constructed and transfected into hippocampal neurons with the 5-HT6 vector, after which the length of the primary cilia was measured (Fig. 5b). Cotransfection of ARL13B and 5-HT6 shortened the primary cilia, in comparison with transfection of 5-HT6 alone (Fig. 5c), indicating that ARL13B recovered cilia elongation induced by overexpression of 5-HT6. Total protein expression of ARL13B was not influenced by 5-HT6 overexpression or knock-down (Fig. 5d). 5-HT6 mutants D72A and F69L + T70I + D72A altered the activity of cyclic adenosine monophosphate (cAMP). When 5-HT concentrations in HEK293T cells were increased gradually, cells with the D72A and F69L + T70I + D72A mutations showed a reduced cAMP concentration in comparison with that of cells with WT 5-HT6 (Fig. 5e). These results showed that ARL13B and downstream cAMP activation were involved in 5-HT6 regulation of ciliary length.

### 5-HT6 altered axonal length and AIS morphology

Interestingly, ankyrin G (AnkG), a typical marker for the AIS [24], was translocated to the primary cilia of hippocampal neurons with overexpression of 5-HT6 (Fig. 4c). Mutating 5-HT6 did not restore the AIS localization of AnkG (Additional file 5: Figure S5A). To determine whether 5-HT6 affects total protein levels of AnkG, we measured their expression levels in HEK293T cells with 5-HT6 overexpression or knock-down. We found that 5-HT6 had no influence on the total protein levels of AnkG (Additional file 5: Figure S5B). Together, these results revealed that 5-HT6 influenced the localization of AnkG without affecting its total protein abundance.

**Fig. 6:**
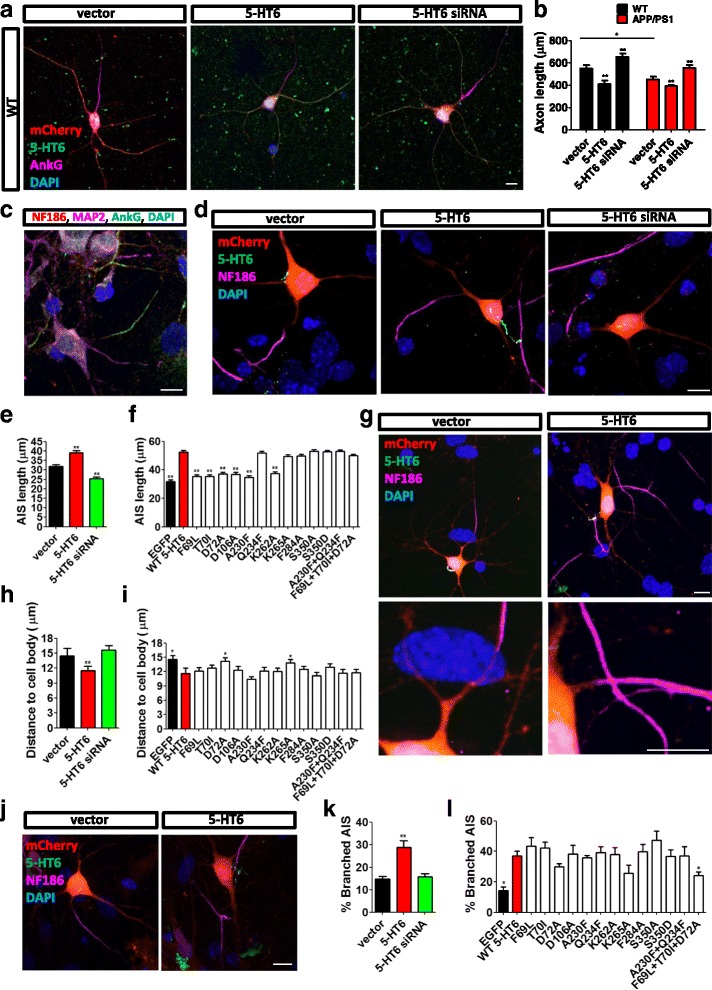
5-HT6 regulated axonal length in hippocampal neurons. (**a**) 5-HT6 regulated axonal length of neurons in WT mice. AnkG (purple), mCherry (red), and DAPI (blue) indicated the axons of hippocampal neurons. (**b**) 5-HT6 influenced axonal length in WT and APP/PS1 mice. Axonal length in APP/PS1 mice was shorter than that of WT mice. Overexpression of 5-HT6 decreased axonal length. Knocking down 5-HT6 increased axonal length. 5-HT6 versus vector in WT, **p < 0.01; 5-HT6 siRNA versus vector in WT, **p < 0.01; 5-HT6 versus vector in APP/PS1, **p < 0.01; 5-HT6 siRNA versus vector in APP/PS1, **p < 0.01; APP/PS1 versus WT in vector, *p < 0.05 (two-way ANOVA: interaction, *F*_2,115_ = 1.05, *p* = 0.35; treatment, *F*_2,115_ = 20.22, *p* < 0.001; gene, *F*_1,115_ = 7.94, *p* < 0.01) (**c**) AnkG indicated the AIS. NF186 (red) and AnkG (green) were markers of the AIS. MAP2 (purple) marked neurons. (**d**) 5-HT6 regulated AIS length. NF186 (purple) indicated the AIS. (**e**) Overexpression of 5-HT6 increased AIS length. Knocking down 5-HT6 decreased AIS length. 5-HT6, **p < 0.01; 5-HT6 siRNA, **p < 0.01 (Kruskal–Wallis test, *p* < 0.001). (**f**) 5-HT6 mutations influenced AIS length. Mutations F69L, T70I, D72A, D106A, A230F, and K262A recovered normal AIS length. EGFP, **p < 0.01; F69L, **p < 0.01; T70I, **p < 0.01; D72A, **p < 0.01; D106A, **p < 0.01; A230F, **p < 0.01; K262A, **p < 0.01 (Kruskal–Wallis test, *p* < 0.001). (**g**) Overexpression of 5-HT6 reduced the distance between the AIS and cell body. (**h**) Overexpression of 5-HT6 decreased the distance between the AIS and cell body. 5-HT6, **p < 0.01 (Kruskal–Wallis test, *p* < 0.001). (**i**) 5-HT6 mutations influenced the distance between the AIS and cell body. Mutations D72A and K265A significantly recovered the decreased distance between the AIS and cell body. EGFP, *p < 0.05; D72A, *p < 0.05; K265A, *p < 0.05 (Kruskal–Wallis test, *p* < 0.01). (**j**) Overexpression of 5-HT6 increased the percentage of neurons with branched AIS. (**k**) High expression of 5-HT6 significantly increased the percentage of neurons with branched AIS. 5-HT6, **p < 0.01 (Kruskal–Wallis test, *p* < 0.001). (**l**) F69L + T70I + D72A mutation restored the percentage of neurons with branched AIS. EGFP, *p < 0.05; F69L + T70I + D72A, *p < 0.05 (Kruskal–Wallis test, *p* < 0.01). All groups were compared with the vector group or EGFP group. Scale bars, 10 μm. All data presented as mean ± SEM (≥3 independent experiments). 5-HT6, serotonin 6 receptor, AIS axon initial segment, AnkG ankyrin-3, DAPI 4',6-diamidino-2-phenylindole

The AIS is the region initiating action potential at the axon. We measured the AIS length with 5-HT6 overexpression. The AIS length was calculated from the 1/3 brightest point near the soma to the 1/3 brightest point far from the soma [25]. Overexpression of 5-HT6 decreased the axonal length in both WT and APP/PS1 mouse neurons, while siRNA against 5-HT6 increased axonal length (Fig. 6a, b). The axonal length was short in APP/PS1 mice, compared with WT mice (Fig. 6b). In addition, 5-HT6 regulated the morphology of AIS in hippocampal neurons. AnkG and AIS marker neurofascin 186 (NF186) were colocalized at the AIS of hippocampal neurons (Fig. 6c). Since 5-HT6 altered AnkG subcellular localization, we used NF186 as the indictor for the AIS. Overexpression of 5-HT6 increased the AIS length, whereas siRNA against 5-HT6 decreased the AIS length (Fig. 6d, e). Mutations F69L, T70I, D72A, D106A, A230F, and K262A recovered the increased AIS length caused by 5-HT6 overexpression (Fig. 6f and Additional file 6: Figure S6). The distance between the AIS and cell body was influenced by 5-HT6 as well (Fig. 6g, h). Upregulation of 5-HT6 decreased the distance from the AIS to the cell body. Mutations D72A and K265A significantly recovered the decreased distance between the AIS and cell body (Fig. 6i and Additional file 7: Figure S7). In addition, 5-HT6 also affected the percentage of neurons with branched AIS (Fig. 6j, k). Overexpression of 5-HT6 increased the percentage of neurons with branched AIS. The F69L + T70I + D72A mutation of 5-HT6 reduced the percentage of neurons with branched AIS (Fig. 6l and Additional file 8: Figure S8). These results suggested that 5-HT6 influenced the morphology of the AIS in hippocampal neurons, including the AIS length, the distance from the cell body to the AIS, and the percentage of neurons with branched AIS.

In order to explore the function of changed AIS morphology in neurons, some ion channels specifically located at AIS were tested. Nav 1.2, Nav1.6, and Kv1.1 were located at the AIS. The position pattern of Nav1.2 at AIS was altered (Additional file 9: Figure S9A). Overexpression of 5-HT6 increased the AIS length and percentage of branched AIS marked by Nav1.2, and decreased distance from the AIS to the cell body (Additional file 9: Figure S9B). siRNA of 5-HT6 caused the opposite changes. There was no difference in the location of Nav1.6 with regulation of 5-HT6 (Additional file 9: Figure S9C, D). The position pattern of Kv1.1 was also unaltered (Additional file 9: Figure S9E, F).

**Fig. 7:**
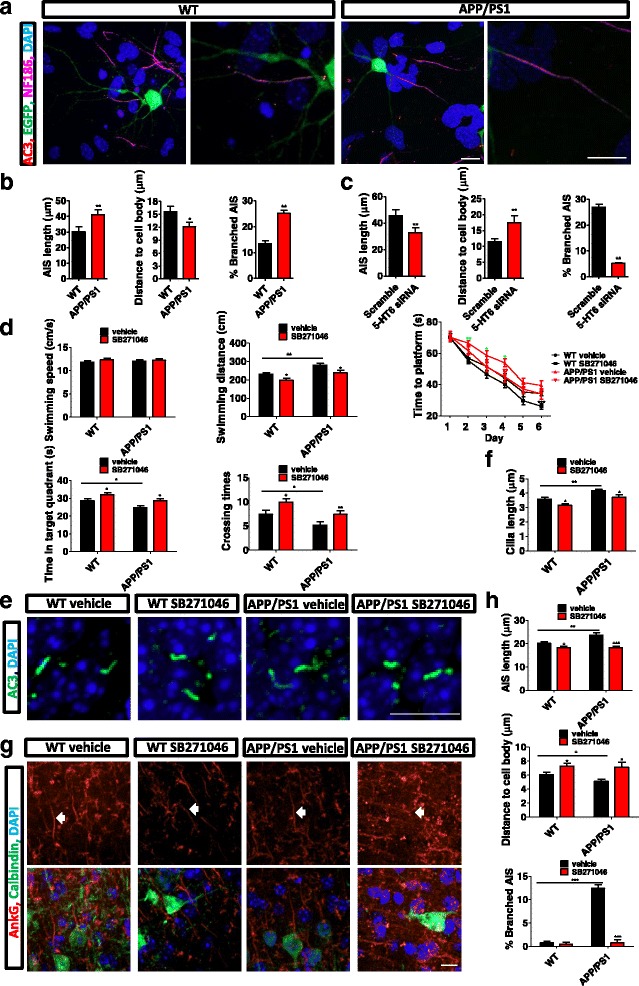
SB271046 restored cognitive impairment of APP/PS1 mice in the Morris water maze test. (**a**) Morphology of the AIS in APP/PS1 and WT mice. AC3 (red) indicated primary cilia. NF186 (purple) indicated the AIS. (**b**) AIS length was significantly increased in APP/PS1 neurons. Distance between the AIS and cell body was reduced in APP/PS1 neurons. Percentage of branched AIS increased in APP/PS1 neurons. AIS length, **p < 0.01 (Mann–Whitney *U* test); distance to cell body, *p < 0.05 (Mann–Whitney *U* test); branched AIS, **p < 0.01 (Mann–Whitney *U* test). (**c**) 5-HT6 siRNA reversed AIS morphological changes in APP/PS1 neurons. AIS length, **p < 0.01 (Mann–Whitney *U* test); distance to cell body, **p < 0.01 (Mann–Whitney *U* test); branched AIS, **p < 0.01 (Mann–Whitney *U* test). (**d**) 5-HT6 antagonist SB271046 had no effect on swimming speed. SB271046 decreased swimming distance and time to reach the platform, and increased time spent in the target quadrant and number of crossings. Compared with WT mice, APP/PS1 mice had obvious cognitive impairment, containing longer swimming distance and time to platform and short time in target quadrant and number of crossings. Swimming speed (two-way ANOVA: interaction, *F*_1,104_ = 0.09, *p* = 0.77; treatment, *F*_1,104_ = 0.06, *p* = 0.81; gene, *F*_1,104_ = 1.38, *p* = 0.24). Swimming distance: vehicle versus SB271046 in WT, *p < 0.05; vehicle versus SB271046 in APP/PS1, *p < 0.05; WT versus APP/PS1 in vehicle, **p < 0.01 (two-way ANOVA: interaction, *F*_1,104_ = 0.11, *p* = 0.74; treatment, *F*_1,104_ = 11.15, *p* < 0.01; gene, *F*_1,104_ = 16.99, *p* < 0.001). Time to platform (three-way ANOVA: day, *F*_5,536_ = 118.8, *p* < 0.001; treatment, *F*_1,536_ = 17.62, *p* < 0.001; gene, *F*_1,536_ = 29.06, *p* < 0.001; day × treatment, *F*_5,536_ = 1.08, *p* = 0.37; day × gene, *F*_5,536_ = 1.04, *p* = 0.39; treatment × gene, *F*_1,536_ = 0.2, *p* = 0.65; day × treatment × gene, *F*_5,536_ = 0.33, *p* = 0.9). Time in target quadrant: vehicle versus SB271046 in WT, *p < 0.05; vehicle versus SB271046 in APP/PS1, *p < 0.05; WT versus APP/PS1 in vehicle, *p < 0.05 (two-way ANOVA: interaction, *F*_1,101_ = 0.02, *p* = 0.88; treatment, *F*_1,101_ = 9.64, *p* < 0.01; gene, *F*_1,101_ = 9.88, *p* < 0.01). Crossing times: vehicle versus SB271046 in WT, *p < 0.05; vehicle versus SB271046 in APP/PS1, **p < 0.01; WT versus APP/PS1 in vehicle, *p < 0.05 (two-way ANOVA: interaction, *F*_1,104_ = 0.02, *p* = 0.88; treatment, *F*_1,104_ = 9.76, *p* < 0.01; gene, *F*_1,104_ = 10.47, *p* < 0.01). (**e**) SB271046 decreased cilia length after water maze of 7 days in vivo. (**f**) SB271046 restored the longer cilia of APP/PS1 mice. Vehicle versus SB271046 in WT, *p < 0.05; vehicle versus SB271046 in APP/PS1, *p < 0.05; WT versus APP/PS1 in vehicle, **p < 0.01 (two-way ANOVA: interaction, *F*_1,268_ = 0.05, *p* = 0.82; treatment, *F*_1,268_ = 9.25, *p* < 0.01; gene, *F*_1,268_ = 15.49, *p* < 0.001). (**g**) SB271046 recovered AIS morphology in vivo. AnkG indicated AIS of neuron. Arrows indicate AIS. Calbindin indicated neuron. (**h**) SB271046 decreased AIS length, increased distance from AIS to cell body, and deceased percentage of branched AIS of neuron. AIS length: vehicle versus SB271046 in WT, *p < 0.05; vehicle versus SB271046 in APP/PS1, ***p < 0.001; WT versus APP/PS1 in vehicle, **p < 0.01 (two-way ANOVA: interaction, *F*_1,223_ = 5.37, *p* < 0.05; treatment, *F*_1,223_ = 23.45, *p* < 0.001; gene, *F*_1,223_ = 4.91, *p* < 0.05). Distance to cell body: vehicle versus SB271046 in WT, *p < 0.05; vehicle versus SB271046 in APP/PS1, *p < 0.05; WT versus APP/PS1 in vehicle, *p < 0.05 (two-way ANOVA: interaction, *F*_1,222_ = 0.86, *p* = 0.36; treatment, *F*_1,222_ = 12.15, *p* < 0.001; gene, *F*_1,222_ = 4.96, *p* < 0.05). Branched AIS: vehicle versus SB271046 in WT, *p* = 0.79; vehicle versus SB271046 in APP/PS1, ***p < 0.001; WT versus APP/PS1 in vehicle, ***p < 0.001 (two-way ANOVA: interaction, *F*_1,8_ = 87.61, *p* < 0.001; treatment, *F*_1,8_ = 95.26, *p* < 0.001; gene, *F*_1,8_ = 94.72, *p* < 0.001). Scale bars, 10 μm. All data presented as mean ± SEM (≥3 independent experiments). 5-HT6, serotonin 6 receptor, AIS axon initial segment, DAPI 4',6-diamidino-2-phenylindole, EGFP enhanced green fluorescent protein

The AIS of APP/PS1 mouse neurons showed changes similar to those caused by overexpression of 5-HT6 in WT hippocampal neurons (Fig. 7a). The AIS length of APP/PS1 neurons increased significantly in comparison with that of WT mice, while the distance between the AIS and cell body was shorter (Fig. 7b). Moreover, the percentage of hippocampal neurons with branched AIS was higher in APP/PS1 mice than in WT mice (Fig. 7b). siRNA to 5-HT6 successfully recovered the morphologies in APP/PS1 neurons almost to the WT level (Fig. 7c). Together, these results showed that the upregulated level of 5-HT6 was associated with axonal length and AIS morphology alterations in APP/PS1 mice.

### 5-HT6 antagonist SB271046 restored cognitive impairment in APP/PS1 mice

The typical behavior defect of APP/PS1 mice is cognitive impairment. The Morris water maze test was applied to assess cognition after APP/PS1 mice were administered SB271046, a 5-HT6 antagonist. The APP/PS1 mice received 10 mg/kg SB271046 every day for 7 days by intraperitoneal injection. The control group received 0.9% NaCl in the same manner and on the same schedule. The concentration of SB271046 in serum of WT mice increased gradually (Additional file 10: Figure S11C). APP/PS1 mice had typical cognitive impairment, embodied in the longer swimming distance to platform, longer time to platform, shorter time in target quadrant, and fewer times of crossing the original platform (Fig. 7d). The swimming speed of SB270146-treated mice was not different between APP/PS1 and WT mice (Fig. 7d). SB271046 decreased the swimming distance and time to platform, and increased time in the target quadrant and crossing times (Fig. 7d). After the water maze test, the cilia length and AIS morphology of mice were calculated. SB271046 decreased cilia length in APP/PS1 and WT mice (Fig. 7e, f). AIS morphology was recovered by SB271046 (Fig. 7g, h). SB 271046 decreased the AIS length and percentage of branched AIS, and increased the distance between the AIS and cell body (Fig. 7h). Also, siRNA of 5-HT6 increased arborization of dendrites in APP/PS1 and WT mice in cultured neurons. The arborization of dendrites of neurons in APP/PS1 mice was lower than that of WT mice in vitro (Additional file 11: Figure S10A-B). SB271046 also improved arborization of dendrites of APP/PS1 and WT mice in vivo (Additional file 11: Figure S10C, D).

In addition, the Y maze and fear conditioning were used to tested the function of 5-HT6 in cognition. SB271046 increased the percentage of correct times in APP/PS1 and WT mice (Additional file 10: Figure S11A). The percentage of correct times which APP/PS1 mice chose was lower than that of WT mice. Fear conditioning was a typical test to assess the memory of mice. SB271046 enhanced duration of freezing in APP/PS1 and WT mice (Additional file 10: Figure S11B). The duration of freezing time in APP/PS1 mice was lower than that of WT mice. Collectively, results indicated that 5-HT6 antagonist SB271046 recovered the cognitive impairment of APP/PS1 mice.

## Discussion

Previous studies showed that 5-HT6 is located at the primary cilia, where it plays an important role [22]. Therefore, we suspected that 5-HT6 may influence cognition in AD patients by regulating the primary cilia function. The results of our study showed that overexpression of 5-HT6 increased the length of primary cilia. Meanwhile, 5-HT6 was upregulated in the hippocampus of APP/PS1 mice. 5-HT6, a G protein coupled receptor, not only regulated the cilia length and branch but also had an effect on the location of ARL13B and AnkG in primary cilia. 5-HT3A, which belongs to the Cys-loop superfamily of ligand-gated ion channels, also affected the cilia length. Both 5-HT6 and 5-HT3A are 5-HT receptors. This indicated that 5-HT receptors might cause the imbalance of some proteins and compromise ciliary signaling. The disorder of proteins in cilia could impair the ability of cilia to detect and respond to signaling in the local extracellular environment. As a result, ciliary function was influenced in APP/PS1 mice.

In addition, 5-HT6 regulated AIS morphology and axonal length. Compared to WT mice, the axonal length was shorter in APP/PS1 mice. The synapse, the function of which is transmitting a signal to the next neuron, is at the end of the axon. Therefore, the shorter axonal length in APP/PS1 mice might affect synaptic signal transmission in a distant position. In hippocampal neurons of APP/PS1 mice, the AIS was elongated, showed a greater degree of branching, and was closer to the cell body. There are many ion channels anchored at the AIS of neurons. The longer AIS length in APP/PS1 mice might influence the location of these ion channels, and then the initiation of action potential might be affected. Besides, the longer AIS might also affect protein transporting into the axon, which would lead to the variation of axonal length. The decreased axonal length and increased AIS length were comprehensive and complex results in APP/PS1 mice and the concrete mechanism should be explored in future experiments.

Interestingly, our finding that ciliary length was increased in APP/PS1 mice differs from that of a previous study on 3xAD transgenic mice, in which cilia length was reduced in hippocampal dentate granule cells [26]. We speculated that three factors might underlie this difference. Different animal models of AD were used in each study. 3xAD-transgenic mice harbor APP_Swe_, PS1_M148V_, and tau_P301L_, while the transgenic mice used in this study harbor APP_swe_ and PS1_ΔE9_. The use of different models of AD may lead to differing results. In addition, different types of cell were assessed in each study. The study using 3xAD-transgenic mice only measured the length of primary cilia in dentate granule cells, while cilia length was measured in dentate granule cells and pyramidal neurons in CA1 and CA3 in our study. Finally, female 3xAD-transgenic mice were used in their study, and male APP/PS1 mice were tested in our experiments. Different gender of the mice might also affect results. The reason for the differences of cilia length among animal models of AD merits further study.

Typical symptoms of patients with AD include severe deficits in learning and memory. Several studies have shown that 5-HT6 antagonists can improve learning and memory ability [27]. In this study, we aimed to understand the mechanism through which 5-HT6 antagonists improve cognition in patients with AD. Numerous convincing studies have shown that there is a strong correlation between the primary cilia and cognition, indicating that 5-HT6 may affect cognition through the primary cilia. Children with primary ciliary dyskinesia, a typical ciliopathy due to structure and functional abnormalities of respiratory cilia, showed significant behavioral and social competence problems [28]. The cognitive functioning and memory performances of patients with Bardet-Biedl Syndrome (BBS), which is also a kind of ciliopathy, was due to hippocampal dysgenesis [29]. Joubert syndrome (JS) in patients with intellectual disability is also related to primary cilia [30]. Primary cilia deficiency can lead to ciliopathies, including Joubert syndrome (JBTS), polycystic kidney disease, developmental disorders, cognitive impairment, and cognitive damage [21]. Interfering with the normal function of BBS complex delays development and leads to mental retardation and learning impairment [31]. SB399885, another antagonist of 5-HT6, could shorten primary cilia of striatal neurons after 24 hours in culture [32]. It also reversed the age-dependent deficit of rats in the water maze test [33]. In mice, interfering with genes required for proper cilia function delays spatial learning and recognition [34]. Mutation of SSTR3, which is located at the primary cilia, impairs hippocampal LTP in mice [35, 36]. AC3, which was located at primary cilia, was required for novel object learning and extinction of contextual memory [37]. Our water maze test, Y maze, and fear condition results showed that 5-HT6 antagonist SB271046 recovered cognitive impairment in APP/PS1 mice, indicating that 5-HT6 has a close relationship with the cognition of APP/PS1 mice. Meanwhile, our results showed that primary cilia length was recovered by SB271046 in behavioral experiments, which strongly suggested that 5-HT6 might affect the cognition through regulating the primary cilia. The more specific inner mechanism deserves future study.

## Conclusions

Our data showed that 5-HT6 regulated ciliary length through ARL13B, influenced the function of neuronal primary cilia, affected the AIS morphology and axonal length in APP/PS1 mice. Besides, SB271046, 5-HT6 antagonist, could recover the cognitive impairment of APP/PS1 mice. Based on our results and those of reported studies, we suggest that 5-HT6 plays a critical role in AD development through regulating the morphology and function of neuronal primary cilia, which is possibly related to the AIS and axon alterations in AD development.

## Additional files

Additional file 1: Figure S1. Showing 5-HT6 located at the primary cilia of hippocampal neurons. (**A**) Endogenous 5-HT6 located at the primary cilia of neurons. ARL13B and SSTR3 were markers of primary cilia. MAP2 was the neuronal marker. γ-tubulin was the marker of the centrosome from which primary cilia grow. (**B**) Exogenous 5-HT6, which was expressed from 5-HT6-GFP vector, located at the primary cilia of neurons. ARL13B and IFT88 were markers of primary cilia. Scale bars, 10 μm. (DOC 8716 kb)

Additional file 2: Figure S2. Showing siRNAs of 5-HT6 and 5-HT3A decreased cilia length. (**A**) Both 5-HT6 and 5-HT3A affected cilia length in hippocampal neurons. AC3 indicated cilia. (**B**) siRNA of 5-HT6 and 5-HT3A reduced cilia length. 5-HT3A-siRNA, **p < 0.01; 5-HT6-siRNA, *p < 0.05 (Kruskal–Wallis test, *p* < 0.001). Scale bars, 10 μm. All data presented as mean ± SEM. (DOC 4600 kb)

Additional file 3: Figure S3. Showing the effect of 5-HT6 on cilia length in excitatory and inhibitory neurons. (**A**) 5-HT6 regulated cilia length in excitatory neurons. 5-HT6 indicated cilia. CamkII was the marker of excitatory neuron. (**B**) Overexpression of 5-HT6 increased cilia length. siRNA of 5-HT6 reduced cilia length. 5-HT6, ***p < 0.001; 5-HT6 siRNA, *p < 0.05 (Kruskal–Wallis test, *p* < 0.001). (**C**) 5-HT6 regulated cilia length in inhibitory neurons. Gad was the maker of inhibitory neuron. 5-HT6 indicated cilia. (**D**) Cilia length increased by overexpression of 5-HT6 and decreased by siRNA of 5-HT6. 5-HT6, ***p < 0.001; 5-HT6 siRNA, *p < 0.05 (Kruskal–Wallis test, *p* < 0.001). Scale bars, 10 μm. All data presented as mean ± SEM. (DOC 4724 kb)

Additional file 4: Figure S4. Showing 5-HT6 mutations influenced the number of ciliary branches and the percentage of neurons with branched cilia. (**A**) Mutations D72A, D106A, and F69L + T70I + D72A recovered the number of branches in cilia. Mutations F69L, D72A, D106A, F284A, A230F + Q234F, and F69L + T70I + D72A restored the percentage of neurons with branched cilia. 5-HT6-EGFP indicated primary cilia. (**B**) Sequence information of all overexpression, siRNA, and specific point mutations. Underline indicates sequence after the mutation; () indicates original sequence. Scale bars, 10 μm. (DOC 6514 kb)

Additional file 5: Figure S5. Showing 5-HT6 influenced the location of AnkG between AIS and cilia. (**A**) AnkG (purple) located at the primary cilia following overexpression of 5-HT6 in hippocampal neurons. Mutations of 5-HT6 did not recover the location of AnkG in primary cilia. 5-HT6-EGFP (green) indicated primary cilia. (**B**) 5-HT6 had no effect on total protein level of AnkG in HEK293T cells. Kruskal–Wallis test, *p* = 0.77. β-actin was the internal control. Scale bars, 10 μm. All data presented as mean ± SEM. (DOC 6089 kb)

Additional file 6: Figure S6. Showing 5-HT6 mutations influenced AIS length in hippocampal neurons. Mutations F69L, T70I, D72A, D106A, A230F, and K262A decreased AIS length, compared with that of overexpression of 5-HT6. 5-HT6-EGFP (green) indicated primary cilia. NF186 (purple) was the marker of the AIS. Scale bars, 10 μm. (DOC 6200 kb)

Additional file 7: Figure S7. Showing 5-HT6 mutations influenced the distance between the AIS and cell body in hippocampal neurons. Mutations D72A and K265A restored the distance between the AIS and cell body. 5-HT6-EGFP (green) indicated primary cilia. NF186 (purple) was a marker of the AIS. Scale bars, 10 μm. (DOC 6784 kb)

Additional file 8: Figure S8. Showing 5-HT6 mutations influenced the percentage of cells with branched AIS in hippocampal neurons. Mutations F69L + T70I + D72A decreased the percentage of neurons with branched AIS, compared with that of 5-HT6 overexpression. 5-HT6-EGFP (green) indicated primary cilia. NF186 (purple) was the marker of the AIS. Scale bars, 10 μm. (DOC 7506 kb)

Additional file 9: Figure S9. Showing 5-HT6 regulated AIS morphology marker by Nav1.2. (**A**) 5-HT6 affected the Nav1.2 positioning pattern in the AIS. Nav1.2 was located at the AIS of neurons specifically. (**B**) Overexpression of 5-HT6 increased AIS length and percentage of branched AIS marked by Nav1.2, and decreased distance from AIS to cell body. siRNA of 5-HT6 had the opposite results. AIS length: 5-HT6, *p < 0.05; 5-HT6 siRNA, *p < 0.05 (Kruskal–Wallis test, *p* < 0.01). Distance to cell body: 5-HT6, *p < 0.05; 5-HT6 siRNA, *p < 0.05 (Kruskal–Wallis test, *p* < 0.001). Branched AIS: 5-HT6, *p < 0.05; 5-HT6 siRNA, *p < 0.05 (Kruskal–Wallis test, *p* < 0.01). (**C**) 5-HT6 had no effect on Nav1.6 positioning pattern in AIS. Nav1.6 was located at AIS of neuron specifically. (**D**) There was no difference in positioning pattern of Nav1.6 with regulation of 5-HT6. AIS length (Kruskal–Wallis test, *p* = 0.94), distance to cell body (Kruskal–Wallis test, *p* = 0.59), branched AIS (Kruskal–Wallis test, *p* = 0.44). (**E**) 5-HT6 had no effect on Kv1.1 positioning pattern in AIS. (**F**) Positioning pattern of kv1.1 unaltered. AIS length (Kruskal–Wallis test, *p* = 0.06), distance to cell body (Kruskal–Wallis test, *p* = 0.68), branched AIS (Kruskal–Wallis test, *p* = 0.38). Scale bars, 10 μm. All data presented as mean ± SEM. (DOC 6017 kb)

Additional file 10: Figure S11. Showing SB271046 restored cognitive impairment of APP/PS1 mice. (**A**) SB271046 improved the percentage of correct times in APP/PS1 and WT mice in Y maze. APP/PS1 mice chose more wrong arms, compared with WT mice. Vehicle versus SB271046 in WT, *p < 0.05; vehicle versus SB271046 in APP/PS1, *p < 0.05; WT versus APP/PS1 in vehicle, *p < 0.05 (two-way ANOVA: interaction, *F*_1,34_ = 0.02, *p* = 0.88; treatment, *F*_1,34_ = 11.88, *p* < 0.01; gene, *F*_1,34_ = 7.84, *p* < 0.01). (**B**) SB271046 increased the duration of freezing in APP/PS1 and WT mice in fear conditioning. The duration of freezing of APP/PS1 mice was longer than that of WT mice. Vehicle versus SB271046 in WT, *p < 0.05; vehicle versus SB271046 in APP/PS1, *p < 0.05; WT versus APP/PS1 in vehicle, *p < 0.05 (two-way ANOVA: interaction, *F*_1,33_ = 1.41, *p* = 0.24; treatment, *F*_1,33_ = 11.51, *p* < 0.01; gene, *F*_1,33_ = 15.35, *p* < 0.001). (**C**) Concentration of SB271046 in serum in water maze test. HPLC-MS was used to measure concentration of SB271046 in serum after 2 hours of injecting drug. Concentration of SB271046 was increased gradually from 2 μM on day 1 to 12 μM ion day 7. Kruskal–Wallis test, *p* < 0.01. (DOC 190 kb)

Additional file 11: Figure S10. Showing 5-HT6 affected the arborization of dendrites. (**A**) 5-HT6 influenced arborization of dendrites of excitatory neurons in vitro. CamKII was the maker of excitatory neurons. (**B**) siRNA of 5-HT6 increased the arborization of dendrites in cultured excitatory neuron APP/PS1 and WT mice. The arborization of dendrites was lower in neurons of APP/PS1 mice, compared with WT mice (three-way ANOVA: distance, *F*_18,4465_ = 445.26, *p* < 0.001; treatment, *F*_1,4465_ = 5.3, *p* < 0.05; gene, *F*_1,4465_ = 5.69, *p* < 0.05; distance × treatment, *F*_18,4465_ = 4.85, *p* < 0.001; distance × gene, *F*_18,4465_ = 24.85, *p* < 0.001; treatment × gene, *F*_1,4465_ = 102.84, *p* < 0.001; distance × treatment × gene, *F*_18,4465_ = 6.49, *p* < 0.001). (**C**) 5-HT6 influenced arborization of dendrites of excitatory neuron in vivo. (**D**) SB271046 enhanced arborization of dendrites of APP/PS1 and WT mice. Arborization of dendrites was lower in neurons of APP/PS1 mice, compared with WT mice (three-way ANOVA: distance, *F*_14,4005_ = 59.3, *p* < 0.001; treatment, *F*_1,4005_ = 11.3, *p* < 0.001; gene, *F*_1,4005_ = 18.91, *p* < 0.001; distance × treatment, *F*_14,4005_ = 0.98, *p* = 0.48; distance × gene, *F*_14,4005_ = 1.97, *p* < 0.05; treatment × gene, *F*_1,4005_ = 13.74, *p* < 0.001; distance × treatment × gene, *F*_14,4005_ = 0.88, *p* = 0.59). Scale bars, 10 μm. All data presented as mean ± SEM. (DOC 6890 kb)

## Abbreviations

5-HT6: Serotonin 6 receptor
AC3: Adenylyl cyclase 3
AD: Alzheimer’s disease
AIS: Axon initial segment
AnkG: Ankyrin-3
APP/PS1: Amyloid precursor protein/presenilin 1
ARL13B: ADP-ribosylation factor-like protein 13B
Aβ: Amyloid beta
CamkII: Ca^2+^/calmodulin-dependent protein kinase II
CNS: Central nervous system
DAPI: 4',6-diamidino-2-phenylindole
EGFP: Enhanced green fluorescent protein
FAD: Familial Alzheimer’s disease
Gad: Glutamic acid decarboxylase
IFT88: Intraflagellar transport protein 88
IP: Immunoprecipitation
Kv1.1: Potassium voltage-gated channel subfamily A member 1
MAP2: Microtubule-associated protein 2
Nav1.1: Sodium channel, voltage-gated, type I, alpha subunit
Nav1.2: Sodium channel, voltage-gated, type II, alpha subunit
Nav1.6: Sodium channel, voltage gated, type VIII, alpha subunit
NF186: Neurofascin 186
NFT: Neurofibrillary tangles
PFC: Prefrontal cortex
SSTR3: Somatostatin receptor 3
