# Supplementary material for: Serotonin 5-HT6 receptors affect cognition in a mouse model of Alzheimer’s disease by regulating cilia function
Source: Alzheimers Res Ther. 2017 Sep 20;9:76. doi: 10.1186/s13195-017-0304-4 (PMC5607612; doi:10.1186/s13195-017-0304-4)
Supplement: Supplementary file 1 [file 13195_2017_304_MOESM10_ESM.doc]

Fig. S11

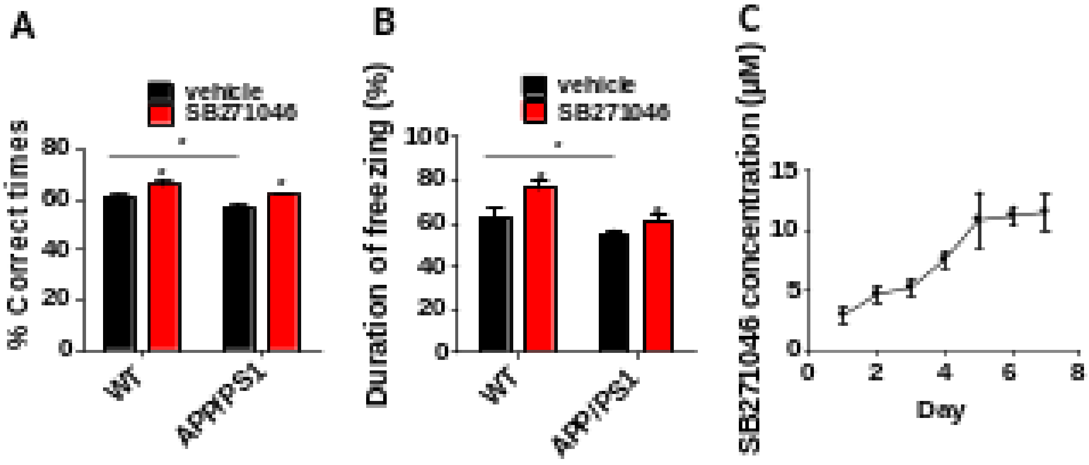

A
B
C
vehicle
SB271046
% Correct times
80
60
40
20
0
WT
APP/PS1
Duration of freezing (%)
100
80
60
40
20
0
SB271046 concentration (μM)
15
10
5
0
2
4
6
8
Day
