# Supplementary material for: Serotonin 5-HT6 receptors affect cognition in a mouse model of Alzheimer’s disease by regulating cilia function
Source: Alzheimers Res Ther. 2017 Sep 20;9:76. doi: 10.1186/s13195-017-0304-4 (PMC5607612; doi:10.1186/s13195-017-0304-4)
Supplement: Supplementary file 2 [file 13195_2017_304_MOESM11_ESM.doc]

Fig. S10

A

WT EGFP | WT 5-HT6-siRNA | APP/PS1 EGFP | APP/PS1 5-HT6-siRNA

CaMKII, EGFP, DAPI

B

C

WT vehicle | WT SB271046 | APP/PS1 vehicle | APP/PS1 SB271046

EGFP, DAPI

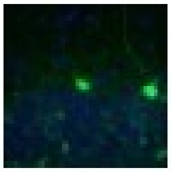

D
