# Supplementary material for: Serotonin 5-HT6 receptors affect cognition in a mouse model of Alzheimer’s disease by regulating cilia function
Source: Alzheimers Res Ther. 2017 Sep 20;9:76. doi: 10.1186/s13195-017-0304-4 (PMC5607612; doi:10.1186/s13195-017-0304-4)
Supplement: Supplementary file 3 [file 13195_2017_304_MOESM1_ESM.doc]

Fig. S1

A

MAP2 | 5-HT6 | ARL13B | merge | merge

ARL13B | 5-HT6 | SSRT-3 | DAPI | merge | merge

MAP2 | 5-HT6 | γ-tubulin | DAPI | merge | merge

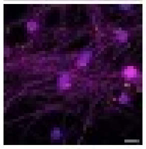

B

DAPI | mCherry | 5-HT6 | ARL13B | merge | merge

DAPI | mCherry | 5-HT6 | IFT88 | merge | merge

DAPI | mCherry | 5-HT6 | γ-tubulin | merge | merge
