# Supplementary material for: Serotonin 5-HT6 receptors affect cognition in a mouse model of Alzheimer’s disease by regulating cilia function
Source: Alzheimers Res Ther. 2017 Sep 20;9:76. doi: 10.1186/s13195-017-0304-4 (PMC5607612; doi:10.1186/s13195-017-0304-4)
Supplement: Supplementary file 4 [file 13195_2017_304_MOESM2_ESM.doc]

Fig. S2

A

EGFP | 5-HT1A-siRNA | 5-HT1B-siRNA | 5-HT1D-siRNA | 5-HT1F-siRNA | 5-HT2A-siRNA

5-HT2B-siRNA | 5-HT2C-siRNA | 5-HT3A-siRNA | 5-HT4-siRNA | 5-HT5A-siRNA | 5-HT5B-siRNA

5-HT6-siRNA | 5-HT7-siRNA

AC1 EGFP DAPI

B
