# Supplementary material for: Serotonin 5-HT6 receptors affect cognition in a mouse model of Alzheimer’s disease by regulating cilia function
Source: Alzheimers Res Ther. 2017 Sep 20;9:76. doi: 10.1186/s13195-017-0304-4 (PMC5607612; doi:10.1186/s13195-017-0304-4)
Supplement: Supplementary file 5 [file 13195_2017_304_MOESM3_ESM.doc]

Fig. S3

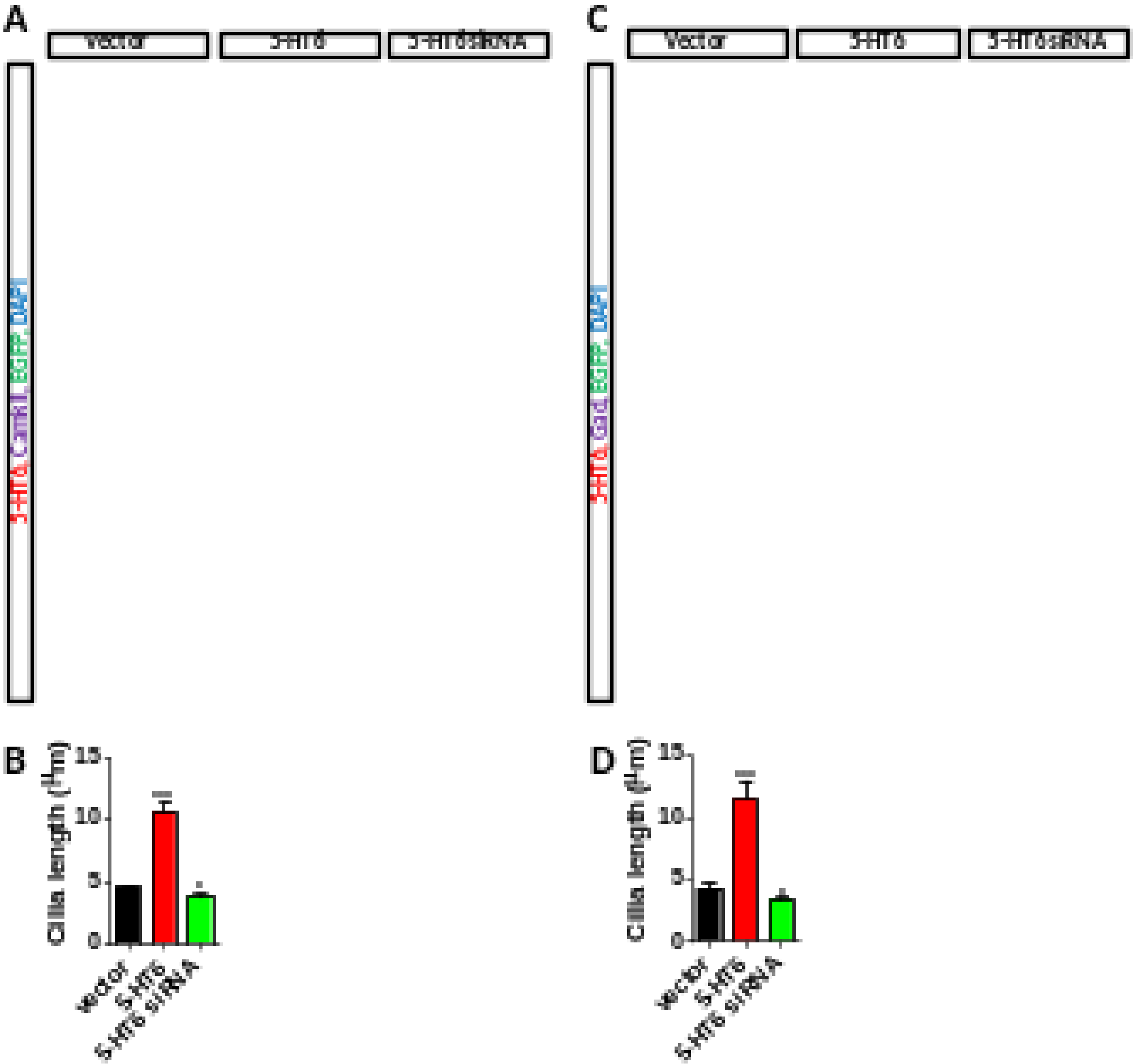
A
Vector
5-HT6
5-HT6siRNA
5-HT6, CamKII, EGFP, DAPI
B
Cilia length (μm)
15
10
5
0
vector
5-HT6
5-HT6 siRNA
C
Vector
5-HT6
5-HT6siRNA
5-HT6, Gad, EGFP, DAPI
D
Cilia length (μm)
15
10
5
0
vector
5-HT6
5-HT6 siRNA
