# Supplementary material for: Serotonin 5-HT6 receptors affect cognition in a mouse model of Alzheimer’s disease by regulating cilia function
Source: Alzheimers Res Ther. 2017 Sep 20;9:76. doi: 10.1186/s13195-017-0304-4 (PMC5607612; doi:10.1186/s13195-017-0304-4)
Supplement: Supplementary file 6 [file 13195_2017_304_MOESM4_ESM.doc]

Fig. S4

A

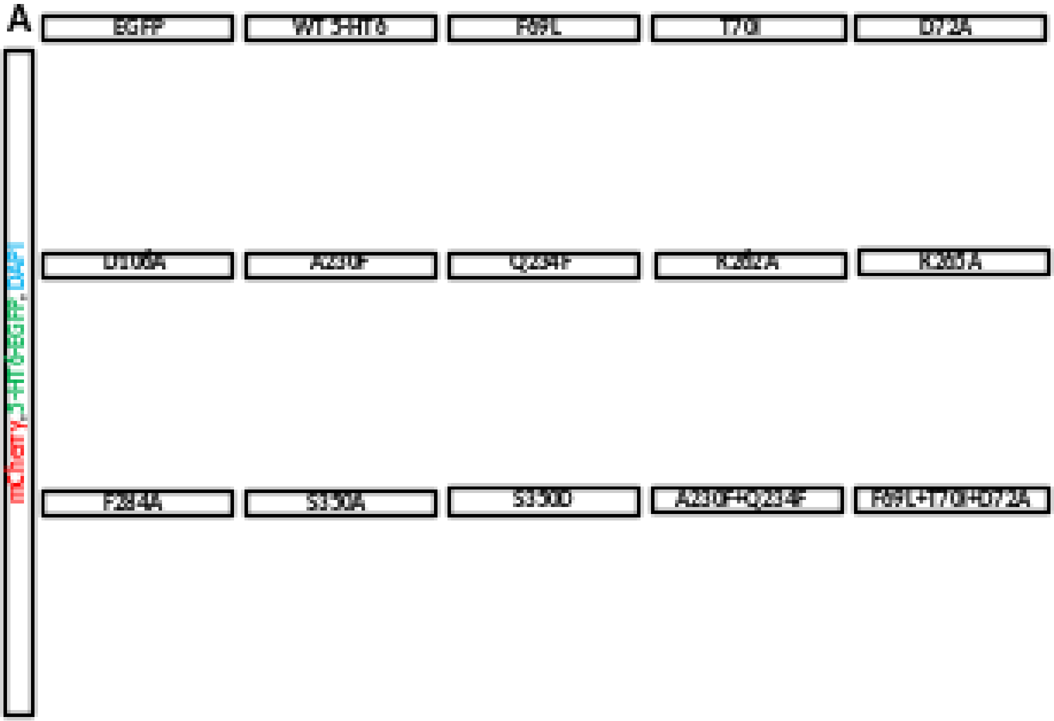

B

Sequence information of 5-HT6

Name	Sequence information
5-HT6	CDS of 5-HT6 in mice
5-HT6 siRNA target sequence	CATGGCTGGGATACTGTAATA
F69L	CCTGGTGTCGCTCTTA（C）ACGTCGGACCT
T70I	TGGTGTCGCTCTTCAT（C）GTCGGACCTG
D72A	CGCTCTTCACGTCGGC（A）CCTGATGGTG
D106A	TTTGGACCGCCTTCGC（A）CGTGATGTGC
A230F	CTCACCACGGGCACGTT（GC）TACGGCTGGCCA
Q234F	ACGGCTACGGCTGGCTTT（CAG）GCCTTGGAAA
K262A	ACCAAGCATAGCAGGGC（AA）GGCCTTGAAG
K265A	AGCAGGAAGGCCTTGGC（AA）GGCCAGCCTG
F284A	ATCCTGCTGAGCATGGC（TT）CTTTGTCACCTGG
S350A	AGCCCCGCCTCCCCCG（T）CCATGTGGACCT
S350D	AGCCCCGCCTCCCCCGA（TC）CATGTGGACCT
