# Supplementary material for: Serotonin 5-HT6 receptors affect cognition in a mouse model of Alzheimer’s disease by regulating cilia function
Source: Alzheimers Res Ther. 2017 Sep 20;9:76. doi: 10.1186/s13195-017-0304-4 (PMC5607612; doi:10.1186/s13195-017-0304-4)
Supplement: Supplementary file 7 [file 13195_2017_304_MOESM5_ESM.doc]

Fig. S5

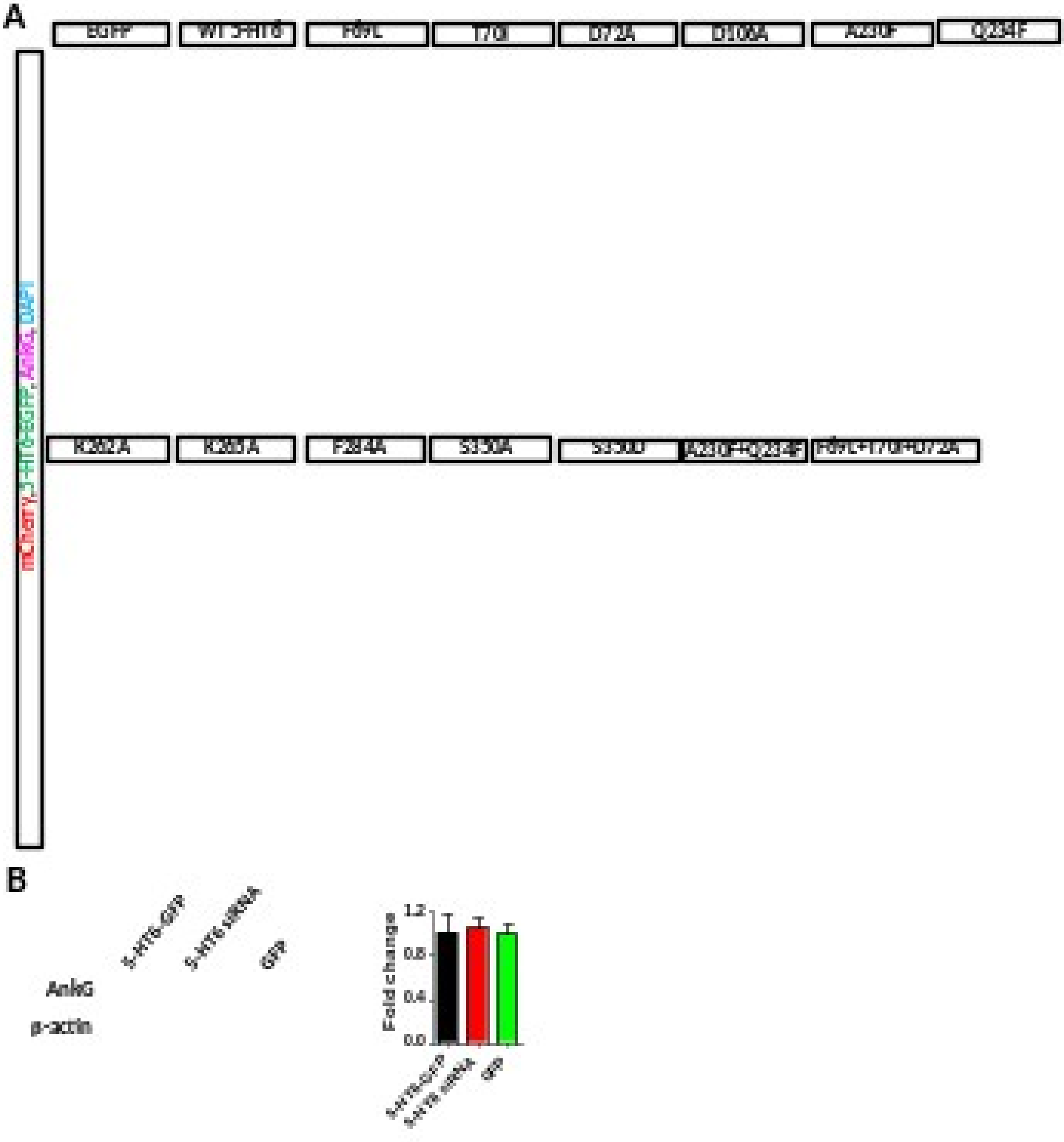
A
EGFP
D72A
D106A
A230F
Q234F
K262A
F284A
S330A
S330D
A230F+Q234F
mCherry, 5-HT6-EGFP, AnkG, DAPI
B
5-HT6-GFP
5-HT6 siRNA
GFP
AnkG
β-actin
Fold change
1.2
0.8
0.4
0.0
