# Supplementary material for: Serotonin 5-HT6 receptors affect cognition in a mouse model of Alzheimer’s disease by regulating cilia function
Source: Alzheimers Res Ther. 2017 Sep 20;9:76. doi: 10.1186/s13195-017-0304-4 (PMC5607612; doi:10.1186/s13195-017-0304-4)
Supplement: Supplementary file 8 [file 13195_2017_304_MOESM6_ESM.doc]

Fig. S6

EGFP	WT 5-HT6	F69L	T70I	D72A	D106A	A230F	Q234F

K262A	K265A	F284A	S350A	S350D	A230F+Q234F	F69L+T70I+D72A

mCherry, 5-HT6-EGFP, [illegible], DAPI
