# Supplementary material for: Serotonin 5-HT6 receptors affect cognition in a mouse model of Alzheimer’s disease by regulating cilia function
Source: Alzheimers Res Ther. 2017 Sep 20;9:76. doi: 10.1186/s13195-017-0304-4 (PMC5607612; doi:10.1186/s13195-017-0304-4)
Supplement: Supplementary file 9 [file 13195_2017_304_MOESM7_ESM.doc]

Fig. 57

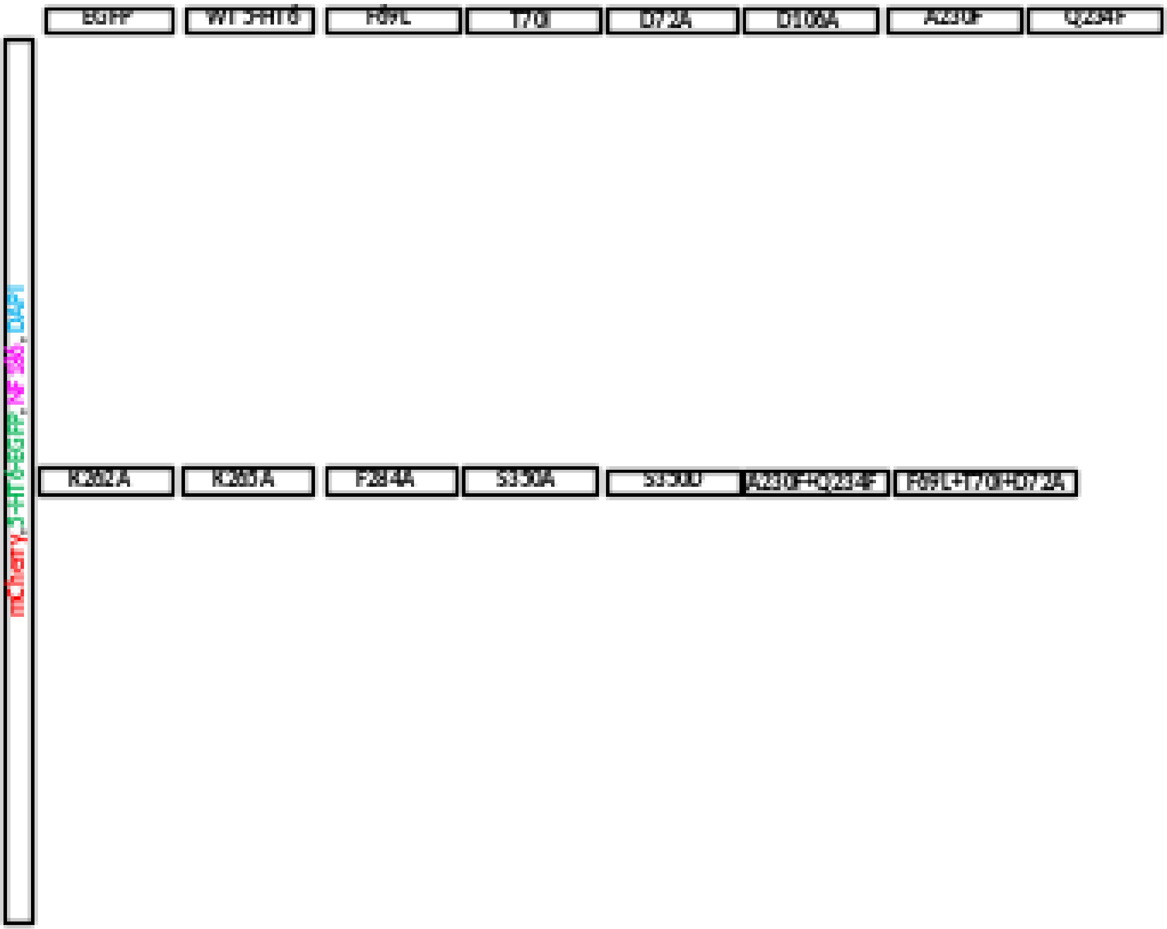
EGFP
WT 5-HT6
F69L
T70I
D72A
D106A
A230F
Q234F
K262A
K265A
F284A
S350A
S350D
A230F+Q234F
F69L+T70I+D72A
mCherry, 5-HT6-EGFP, NF 186, DAPI
