# Supplementary material for: Serotonin 5-HT6 receptors affect cognition in a mouse model of Alzheimer’s disease by regulating cilia function
Source: Alzheimers Res Ther. 2017 Sep 20;9:76. doi: 10.1186/s13195-017-0304-4 (PMC5607612; doi:10.1186/s13195-017-0304-4)
Supplement: Supplementary file 10 [file 13195_2017_304_MOESM8_ESM.doc]

Fig. 58

EGFP | WT 5-HT6 | H69L | T70I | D72A | D106A | A230F | Q234F

mCherry, 5-HT6-EGFP, NF 160, DAPI

K262A | K265A | F284A | S330A | S330D | A230F+Q234F | H69L+T70I+D72A
