# Supplementary material for: Serotonin 5-HT6 receptors affect cognition in a mouse model of Alzheimer’s disease by regulating cilia function
Source: Alzheimers Res Ther. 2017 Sep 20;9:76. doi: 10.1186/s13195-017-0304-4 (PMC5607612; doi:10.1186/s13195-017-0304-4)
Supplement: Supplementary file 11 [file 13195_2017_304_MOESM9_ESM.doc]

Fig. S9

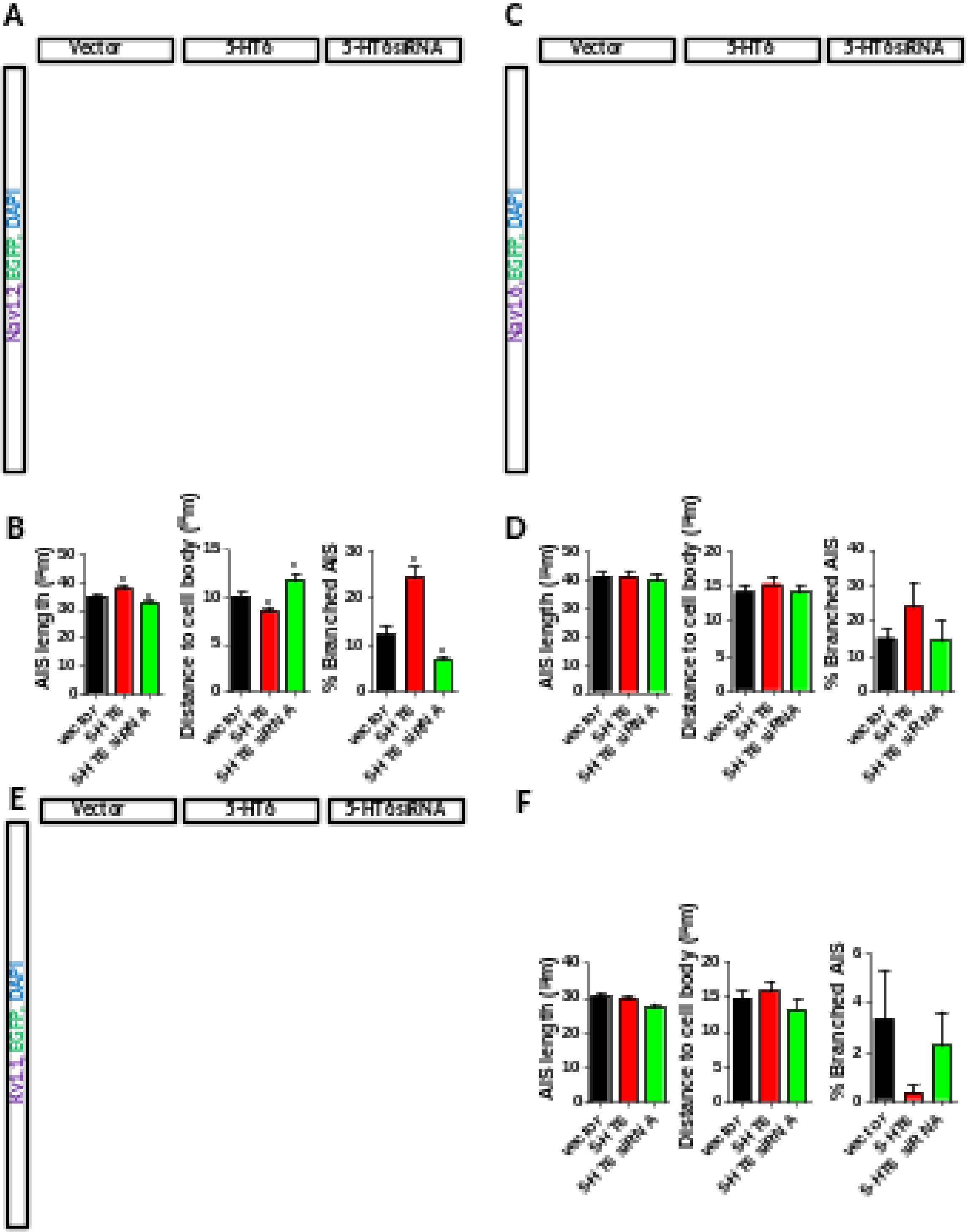
A
Vector
5-HT6
5-HT6siRNA
Nav1.2, EGFP, DAPI
C
Vector
5-HT6
5-HT6siRNA
Nav1.6, EGFP, DAPI
B
AIS length
Distance to cell body
% Branched AIS
D
AIS length
Distance to cell body
% Branched AIS
E
Vector
5-HT6
5-HT6siRNA
Kv1.1, EGFP, DAPI
F
AIS length
Distance to cell body
% Branched AIS
